# Development of an Obesity Information Diagnosis Model Reflecting Body Type Information Using 3D Body Information Values

**DOI:** 10.3390/s22207808

**Published:** 2022-10-14

**Authors:** Changgyun Kim, Sekyoung Youm

**Affiliations:** Department of Industrial and Systems Engineering, Dongguk University, Seoul 04620, Korea

**Keywords:** obesity, body mass index, feature extraction, diagnosis, 3D body

## Abstract

This study uses various body values (length, circumference, and volume) that can be derived from 3D data to determine variables and areas that substantially affect obesity and suggests guidelines for diagnosing obesity that are more elaborate than existing obesity indices. Body data for 170 participants (87 men and 73 women aged 20–30 years) are collected for the chest, abdomen, hips, and arms/legs. A 3D scanner, which can produce accurate body point results, and dual-energy X-ray (DEXA), which can accurately determine the fat percentage, are used to derive fat rates for each body part. The fat percentage and total fat percentage for each body part are used as learning data. For the derived data, the eigenvalue for each body part is derived using a principal component analysis, and the following four clusters are created for each part: underweight, normal, overweight, and obese. A comparison with the obesity index, which diagnoses obesity based on the cluster model, showed that the accuracy of the model proposed in this study is higher at 80%. Therefore, this model can determine the body information necessary for accurate obesity diagnosis and be used to diagnose obesity with greater accuracy than obesity indices without a body fat measurement machine such as DEXA.

## 1. Introduction

The obese population is rapidly increasing due to dietary changes caused by economic growth and the increase in single-person households that regularly order food deliveries [1,2]. According to the WHO, obesity is a serious problem worldwide. Its prevalence has increased rapidly since the 1970s, having increased by 100% compared to in the 2000s [3]. In 2003, obesity was selected as one of the major diseases to be managed by the WHO. This is because obesity causes various chronic diseases. For example, if obesity is not recognized or properly managed, the probability of developing diseases, such as cardiovascular diseases, increases [4]. Therefore, the prevention and management of obesity worldwide is an important issue. As a result, policies are being implemented to prevent obesity in various countries. Overseas, nutrition guides, exercise locations, action plans, and physical activity programs are provided in each city, and obesity prevention strategies have been presented by an international anti-obesity organization [5]. In Korea, the National Obesity Prevention Policy Establishment and Health Promotion Plan 2020 (HP 2020) [6] have been implemented, and obesity projects and related policies are sporadically applied in various institutions, but it is difficult to determine the extent of obesity and implement prevention strategies without an accurate obesity diagnosis.

Methods for diagnosing obesity include the diagnosis of simple body part values, such as height, weight, waist circumference, and hip circumference, using a specific obesity index [7,8] and the use of various methods for diagnosing obesity through body fat thickness, such as bio-electrical impedance analysis (BIA) [9] and dual-energy X-ray (DEXA) [10]. However, obesity diagnosis methods cannot derive accurate obesity information using simple body values [11], whereas obesity diagnosis using expensive equipment can derive accurate obesity information but has limitations in terms of time and cost that require continuous management [12]. Because of these limitations, the obesity index, which can derive obesity information through simple body values, is generally used to diagnose obesity. However, existing obesity indices cannot judge body circumference and volume information, so body characteristics such as body type and muscle mass cannot be considered. It is difficult to accurately diagnose obesity through measurement information that does not consider body type information. In addition, even if body information is known, the measurers do not know which types of body information have important effects on obesity. Therefore, in this study, obesity information was derived through the body fat rate obtained from DEXA, and based on this, we aimed to establish a more accurate obesity standard than that provided using the existing body index by considering body length, circumference, and volume information. Through established obesity standards, a model was created that can derive characteristics for each body part and diagnose obesity through circumference and volume, which reflect body length and body type information. Obesity standards for each part of the body are set based on the body parts’ characteristics, and through the obesity diagnosis model, users can use body information to determine which obesity group they belong to. By using the obesity diagnosis model provided in this study, it is possible to diagnose obesity with a similar accuracy level without the need for expensive obesity diagnosis equipment and using only body length, circumference, and volume information. Further, the criteria for an accurate obesity diagnosis can be determined by region.

## 2. Literature Review

### 2.1. Obesity Diagnosis Method

Obesity is becoming a serious social issue, and research on the relationship between various diseases and obesity is being actively conducted. Obesity is generally diagnosed through the Body Mass Index (BMI) [13], which is determined by height and weight, and the Circumference and Waist–Hip Ratio (WHR) [14], which uses waist circumference [15]. However, obesity diagnosis using these simple body values is inaccurate, because it is not possible to gain information on the proportions and volume of the body. In addition, athletes have greater volumes of muscle and fat-free mass and may have larger body sizes than normal people despite having less body fat [16]. Thus, a body containing a lot of muscle mass can weigh a lot, and even if it is actually normal, it can be judged as overweight or obese based on the BMI. Therefore, an athlete that is of the same height and weight as a member of the general public can be judged as overweight or obese based on their BMI, despite having a lot of muscle mass and being normal [17] Therefore, various studies have been conducted to accurately diagnose obesity. Hamdy et al. (2006) determined that a clinical diagnosis of visceral fat may be more important than an obesity diagnosis when using BMI to assess the risk of hypertension, arteriosclerosis, and coronary artery disease in both thin and obese people. They analyzed patients using the WHR, focusing on visceral fat accumulation. Measurements were taken using abdominal computed tomography scans, and the total amount of visceral fat and the bottles were determined to be correlated [18]. Maffeis et al. (2008) used the WHR in children to determine metabolic risk. They extracted blood samples from body measurements and intravenous fasting, and the measurements showed that children with large waistlines had higher metabolic rates and greater cardiovascular risks than normal children [19]. However, these studies did not consider other body elements as they only judged single measurements such as waist circumference. However, single-body elements do not provide accurate results for obesity diagnosis. Therefore, Browning et al. (2011) conducted a study to find body values that affect obesity through body information and found a correlation between abdominal fat and magnetic resonance imaging (MRI) and waist circumference (WHtR), and bone density measurements (DEXA) [20]. A total of three groups were created (underweight, overweight, and obese; males: 20 females: 20) and analyzed [21].

In addition, as body volume information is emphasized in the diagnosis of obesity, the concept of the Body Volume Index (BVI) has emerged, in which mass divided by the cube of the height tri-ponderal mass index (TMI) is used to estimate body fat levels. This method is more accurate than the body mass index (BMI) [22]. Peterson et al. (2017) showed that BVI is superior to BMI based on a 3D mass index (TMI = divided by a key cube) [23]. Studies have also been conducted to find optimal obesity characteristics through various obesity derivation methods. Woolcott and Bergman (2018) measured the dimensions of various body parts in 1733 experiments, correlated the results with several variables, such as BMI, and added a variable called “hip joint”. They then determined the impact of this new variable on the obesity information derivation [24]. Hart (2019) devised a simple and accurate measurement of body fat rates for obesity diagnosis. In this study, it can be seen that the degree of obesity in humans varies depending on the body fat rate and muscle information, factors that are part of the human physical condition but cannot be determined by BMI. Therefore, for the determination of accurate obesity information using body fat rates, a new standard for body fat rates (Criterion values of PBF: PBF.DXA) was presented [25].

### 2.2. Importance and Necessity of Accurate Diagnosis of Obesity Information

Various obesity diagnosis methods exist, and equipment such as MRI and DEXA is required to accurately diagnose obesity. However, these devices have significant time and space constraints. Therefore, studies have investigated ways to diagnose obesity using single measurements such as weight, height, and waist circumference. However, these studies do not provide information on body shape and body muscle distribution. If obesity is misdiagnosed, body mismanagement can occur, which not only leads to sarcopenic obesity but can also lead to diseases such as metabolic syndrome. Therefore, researchers have studied methods for diagnosing obesity conveniently and accurately, and based on these studies, methods such as BMI and WHtR have emerged.

If obesity is diagnosed using a single measurement, accurate obesity information and other information cannot be provided. Krakauer and Jesse (2012) showed that BMI has no correlation with body type. In addition, BMI cannot diagnose people with excessive body fat as obese because there are difficulties in measuring body fat, and individuals with high-fat content, i.e., bulky muscle groups, can be misclassified as obese [26]. Wong et al. (2021) also could not determine the presence of proper obesity due to differences in abdominal fat and muscle mass, even when the BMI fell into the normal category [27].

### 2.3. Body Image Distortion

Body image distortion is a problem that many people have, and this distortion is a phenomenon caused by dissatisfaction with the body [28]. Unlike methods that carry out measurements based on subjective perception only, body image distortion measures height and weight objectively and then evaluates a person’s own body by combining the measurer’s subjective perceptions of the body based on this [29,30]. There are the following three main types of body image distortion: distortion that appears when viewed through a 2D image, a distortion that appears by simply judging the body’s index by weight or by an incorrect measurement of the body index, and distortion that appears when comparing the body to those shown in external mass media [31]. When distortion of the body occurs, a person can underestimate their own body. Over- or underestimation may lead to problems such as reckless dieting, eating disorder-induced osteoporosis, anemia, or under-evaluation of disease occurrence due to poor body recognition [32]. Brooks et al. (2016) conducted a survey of 1997 Australian residents that assessed physical discontent and mental and physical health. Related eating disorders and found that 70% of all participants showed dissatisfaction with the body. Increased body discontent was associated with increased problems with mental and physical health, affecting the quality of life and psychological pain [33].

In addition to psychological problems, many people think that their bodies are skinny even though they are obese due to distortions caused by simply judging the body index by weight or by using the wrong measurement methods. Therefore, skinny obesity is a serious problem and can lead to sarcopenic obesity if not managed. Skinny obesity has been associated with a prevalence of metabolic syndrome that is four times higher than that of normal-weight individuals, as well as increases in the prevalence of hyperglycemia and central obesity [34]. As such, skinny obesity has emerged as an important issue, and the risk of skinny obesity and its relationship with the disease are being studied. Batsis et al. (2013) examined the relationships between lean obesity, hypertension, and death, and they divided a total of 31,320 subjects aged 60 or older into three groups based on their body fat ratios through a cohort survey, in which a strong association between high blood pressure and death was found in the high body fat group. In addition, women with skinny obesity were found to have a higher mortality rate than women without skinny obesity [35]. Kapoor et al. (2020) examined the relationship between lean obesity and diabetes and hypertension in 1147 Indians, excluding pregnant women, aged in their 30s to 60s. They found that 31.7% of all survey subjects had lean obesity, and the proportions of diabetes and high blood pressure were higher in the lean obesity group than in the general obese group [36]. When the findings of these studies were examined together, the biggest reason for body image distortion was shown to be problems with the criteria used in the measurement method and the time, cost, and space limitations associated with accurate obesity diagnosis.

### 2.4. Importance of Dimensional Awaresness

Moving from a 2D to a 3D user interface can provide additional information about spatial memory, which gives us more information about 3D perception. According to an experiment conducted in InfoVis 2001, the positions of alphabetic characters shown in three dimensions can be remembered more effectively than those shown in two dimensions. In addition, 3D visualization techniques provide users with more information than 2D data due to their use of 3D data [37]. In another study, orthodontists used cephalograms, which are profile X-rays of lateral skulls and soft tissue, when evaluating skeletal, dental, and soft tissue relationships. The accuracy of perception was determined by generating X-rays in two dimensions and three dimensions, with the 3D method deriving an accuracy that was four to five times higher than that obtained with the 2D method for anatomical distance [38]. Srivastav et al. (2018) created a 3D human behavior and operating room space, involving a dataset of complex operating room environments and various human behaviors, which allows 3D behavior data to be used to recognize more postures than can be achieved with 2D behavior data and posture recognition rates [39]. As such, 3D data have more value than 2D information, which not only increases the accuracy of visual perception but also enables detailed information to be derived. Therefore, more accurate obesity information can be derived by using 3D body information, including information on curves and volume.

Therefore, in this study, body fat rate information for each body part that affects obesity was derived from DEXA body fat rate information for each of the five selected body parts, and criteria for deriving detailed obesity information through length information without body fat information were established.

## 3. Materials and Methods

### 3.1. Data Selection

**Figure 1:**
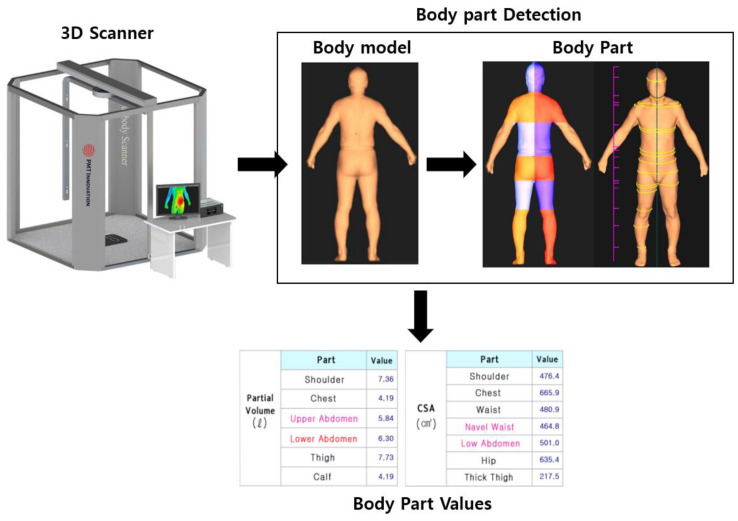
Three-dimensional values derived from the body scan process.

This study generated 3D body shapes through a 3D body scanner to derive body length, circumference, and volume data, and it derived body fat rates for each body part using DEXA. In obesity diagnosis, DEXA can derive the total body fat percentage by obtaining body fat information for each part of the body. It is the most frequently used device and, as such, the obesity diagnosis results obtained with DEXA, which derive accurate results for obesity diagnosis, were designated as the ground truth values, and the accuracy of the obesity diagnosis model was compared using DEXA values. Therefore, based on the body fat rate derived from DEXA, the following four groups were classified: low weight, normal, overweight, and obese. Next, the body fat rates for the arms, legs, chest, stomach, and hips were derived, and we explored which data most affected the overall obesity information. As shown in Table 1, data were collected from a total of 170 people (73 women and 87 men aged 20–30 years) from April to December 2021. With the initial data collection standards, BMI values were derived using height and weight, and groups were recruited as males and females based on BMI. During data collection, people with a BMI of 40 or higher were excluded from the data collection because they were determined to be obese by any obesity diagnostic index. For the experimental group, males (underweight: 20, normal: 24, overweight: 23, obese: 20) and females (underweight: 20, normal: 20, overweight: 18, obese: 15) were collected based on BMI. The accuracy of the measurements was improved by fasting for 3 h before the DEXA inspection. Data were obtained with males wearing bottoms such as tights and females wearing tops and bottoms. The experimenter measured the total body fat rate and the fat rate of each body part through a DEXA and collected body 3D mesh data using a 3D body scanner. The 3D body scanner used in this study was designed based on the ISO-7250 landmark and can derive 60 body index parts. ISO-7250 is a description of the measurement method for creating a body measurement database. It defines landmarks for each body measurement area and serves as a guide for how to measure the body as well as anatomical landmarks, and provides information on an anthropometric basis and on principles of measurement [40] Therefore, based on ISO-7250, measurement standards for each part were defined and body values were derived. Therefore, as shown in Figure 1, the landmark for each part was designated as the ISO-7250 standard, and body values for each part were derived by slicing the 3D body data from the designated standard.

Therefore, DEXA and 3D scanner data from a total of 170 subjects were collected, and variables were derived from each piece of equipment, as shown in Table 1 (DEXA variable data parts). A total of 43 variables were derived from the DEXA measurements, including eight variables for the examiner’s personal information, four variables for measurement time information, 15 variables for the examiner’s total body information, and 16 data points regarding the body fat rate by body part. Among the 43 derived variables, a total of 9 variables, including 6 variables for gender, height, weight variables, and fat rates for each body part, were selected for analysis.

The variables derived through the 3D scanner are shown in Table 2 (3D scanner variable parts). A total of 81 variables, including obesity information and circumference, height, volume, and cross-sectional information for each body part, were derived through each body obesity index derivation method. Among the derived variables, those other than the arms, legs, chest, abdomen, and hips were excluded from the analysis. In addition, to obtain length information among variables, including the length, area, circumference, and volume of the arms, legs, chest, stomach, and hips, five additionally derived length variables were created for each part. Therefore, length values for each body part were derived to obtain length information for each body part. Five length variables were generated, including the chest length = shoulder height-(nostalgia) chest height, waist height = hip height, hip length = hip height, and thigh length = hip height-bottom height, and the previously used height variables were removed. Therefore, a total of 54 variables were finally selected.

**Figure 2:**
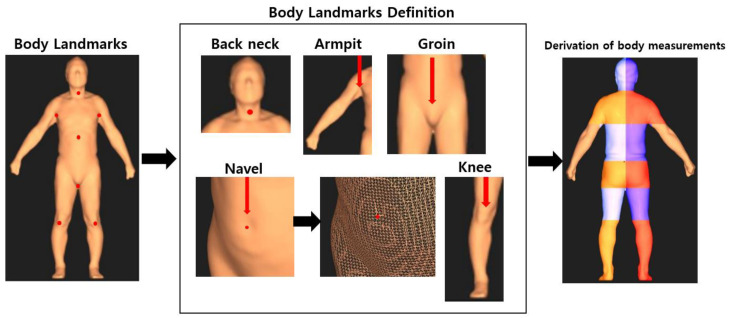
Body point landmark definitions obtained with the 3D scanner.

Definition of landmarks used for deriving body circumference and volume information. As shown in Figure 2, seven automatic measurement points were arbitrarily designated on the back of the object, both armpits, navel, thigh, and both knees on a three-dimensional scanner. At this time, for the arbitrarily designated points, the average dimensions and body designation points of the measurement items corresponding to the body standards were primarily designated. Second, the back of the neck area was designated as the back of the neck as the values came down from the head endpoint where the 3D data began, and the 3D point values from the shoulder to just before the 3D point values increased. For both armpit parts, three-dimensional data were designated as the armpit point by recognizing the part where the values of the points were input in the direction of the arrow starting with the shoulder tip and then disappearing. The navel part was designated as the navel part at the point at which the value decreased in a specific part and returned to the original value, while the point values were input to the waist part. Similar to the shoulder part, certain points continuously entered from the navel point, and the last point before the value disappeared was defined as the thigh. As for the knee part, similar to the navel part, the point value was designated as the knee when there was periodic variation in a specific section. Therefore, through the first and second processes, the 3D scanner derived the circumference and volume processes.

It was assumed that the variables for each body part selected were the values collected for the subjects and that the body type and obesity information for each body part were linearly proportional to the height [41]. Therefore, all body values were divided by the subjects’ heights and normalized through conversion into a ratio with the height. Through this, the body values for each body part were made available in terms of the height information of the subjects.

### 3.2. Data Preprocessing

Based on the total tissue information contained in the DEXA data presented in Table 1 (DEXA variable data parts), the obesity information associated with the body fat percentages of males and females were classified, as shown in Table 2, and participants were divided into four groups based on body weight. Based on DEXA and X-ray data, the body is usually divided into five parts (chest, abdomen, hip, arms, and legs). When deriving body values through these machines, it is possible to classify them in detail, but when detailed inspections for each part are not required, they are broadly divided into five categories [42,43]. Therefore, the obese group was defined according to the total tissue standard, and for a comparison of the relationship between the total body fat percentage and the fat percentage for each body part, five areas—chest tissue, abdomen tissue, hip tissue, arm tissue, and leg tissue—were examined. In addition, the fat percentages of the five body parts (chest, abdomen, hips, arms, and legs) were classified as percentages, as shown in Table 2, because there is no standard for obesity information. The 3D body scanner used in this study generates 3D body data by moving 360° upper and lower body RGB cameras when the user stands in an A-shaped posture. For the generated 3D body data, the 3D scanner automatically designates the landmarks of the body’s curvature and body endpoints based on ISO-7250 and divides the body into the head, chest, abdomen, buttocks, and arms. The thighs and calves are divided into eight parts. For these divided regions, a model was constructed using data from a total of the following five regions: chest, abdomen, buttocks, arms, and legs.

**Figure 3:**
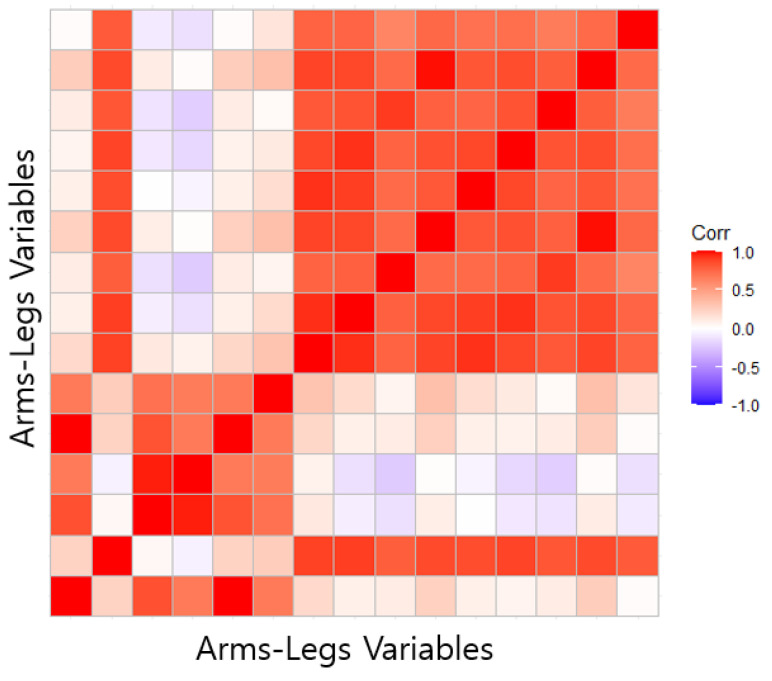
Arms-legs correlation.

Based on the fat percentages of each of the five classified body parts, the variables related to body fat among the body part values derived from the 3D body scanner were grouped together, Among the 54 variables shown in Table 1 (3D scanner variable parts), a correlation analysis was performed on the variables affecting the tissue information (arm, chest, android, gynoid, leg) for each region shown in Table 1 (DEXA variable data parts), and as a result, as shown in Table 3, five variables affecting the branch tissue information were derived, as shown in Table 3. Therefore, a total of five variables were selected for each body part, and variables used for the arms are three-dimensional data The only part that can be extracted from the three-dimensional data is the circumference information. Due to this extraction limitation, only the value of the arm circumference can be derived. The arm circumference value was found to have a strong correlation of 0.9 or more when compared with the leg variables, so these variables were grouped together, and the parts are combined as arms/legs in Figure 3. Therefore, a total of four body part data sets were created.

As shown in Table 3, four body parts (chest, abdomen, hips, arms/legs) that affect the diagnosis of obesity were determined, and a PCA was performed on each of the four body parts for each variable for each part. In this way, factor values for each region were derived through a PCA for each region.

### 3.3. Data Analysis

For determination of the relationships between body fat percentage and total fat percentage for the four body part datasets (chest, abdomen, hips, and arms/legs), the relationships between the variables for each body part and the body fat percentage were investigated. To elucidate the relationship between the four body parts and body fat, classes were created by classifying each male and female by the total body fat percentage. The fat percentage for each body part was classified into four classes based on the total fat percentage, which can be used to judge obesity, as shown in Table 4. Table 4 divides the tissue information by region into four classes for men and women according to the DEXA standard. The class applied the same tissue value for each of the four areas as the criteria for dividing the total tissue into underweight, normal, overweight, and obese categories. As a result of applying the same total tissue value to each of the four regions, categories of Class1→underweight, Class2→normal, Class3→overweight, and Class4→obesity were expressed. Since obesity information about the body parts cannot be determined according to the percentage of body fat like the total fat percentage can, classes were created by dividing the range of body fat percentage for the body fat percentage of each of the four parts. Each class was defined using the criteria of the four classes shown in Table 4, based on the obesity-related criteria and statistical data obtained from a survey of Korean adults and the adult obesity criteria obtained using DEXA Scans [44,45,46]. In addition, data collection and experiments were conducted using two clinical trials and measurement body measurement experts.

Therefore, the variables defined in Table 3 were merged into one dataset based on the four classes classified by fat percentage for each body part, and the principal component analysis (PCA) was used to identify the characteristics of the variables for each body part. The PCA involves finding the principal component of data, identifying multiple characteristics of the distribution of data, reducing features on n vectors to explain the data, calculating the average value of the data $\bar{X}$, and then calculating the covariance matrix *C_x_* of each data point.

Through this, the eigenvector and vi of *C_x_* and the eigenvalue $\lambda_{i}$ were obtained, and the eigenvalues were arranged in the order of largest to smallest. Each eigenvalue refers to the variance value of the data when the axis is transformed in the direction of the corresponding eigenvector, which means that the total sum of $\lambda_{i}$ is the total variance of the data. Therefore, among the obtained eigenvectors and eigenvalues, the entire data set is expressed using the upper t items that satisfy the data expression condition [47].

$$\bar{X}=\frac{1}{n}\overset{n}{\underset{i=1}{\sum }}X_{i}$$

$$C_{x}=\frac{1}{n-1}\overset{n}{\underset{i=1}{\sum }}\left(X_{i}-\bar{X}\right){\left(X_{i}-\bar{X}\right)}^{T}$$

$$V_{T}=\sum_{t}\lambda_{i}\;\left(\lambda_{i}\geq \lambda_{i+1}\right)$$

$$\overset{t}{\underset{i=1}{\sum }}\lambda \geq f_{v}V_{T}$$

$$\left(f_{v}=percentage\;of\;explain\;data\right)$$

The PCA is a method of deriving factors through eigenvectors and providing the meaning of the corresponding class. Unlike a simple classification model, when new information is input, it is a method of classifying the relevant class by using the factors and distance information. The PCA methodology may have a lower classification accuracy than methods using an artificial intelligence model, but it can transparently determine the interactions of factors and performs well in terms of understanding data [48].

**Figure 4:**
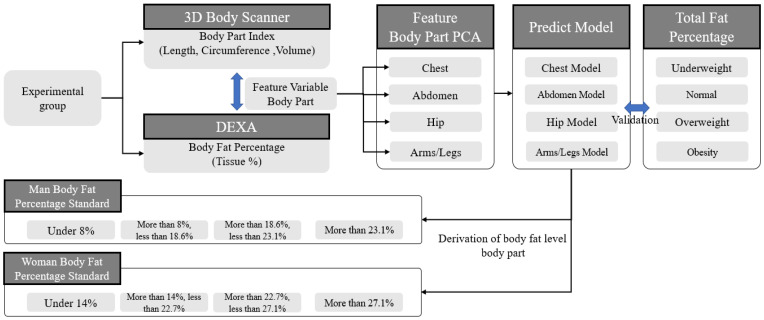
Entire process used in the obesity measurement model.

Therefore, a PCA analysis was performed for each body part dataset based on the body fat percentage range defined in Table 4. As shown in Figure 4, we established the standard body fat percentage for each of the four body parts for men and women; derived the length, circumference, and volume values for each body part assessed by the 3D body scanner; derived the body fat percentage for each body part through a DEXA scan. Therefore, based on the derived body fat percentage, a PCA analysis was performed on the corresponding body value variables for each body part. The obesity classification standard was established through the relationships identified in the PCA results between each region and the total fat percentage. Therefore, the PCA was performed on each of the four body parts (chest, abdomen, hips, and arms/legs), and obesity information for each part was derived through the eigenvalue percentage of variance determined for each PCA.

Through the PCA analysis using male/female body datasets for each of the four body parts, the eigenvalue percentage of variance for each body part was derived, as shown in Table 5. The male and female eigenvalue percentages of variance from Comp 1 to Comp 5 show that Comp 1 and Comp 2 had more than 80% explanatory power for all regions.

**Figure 5:**
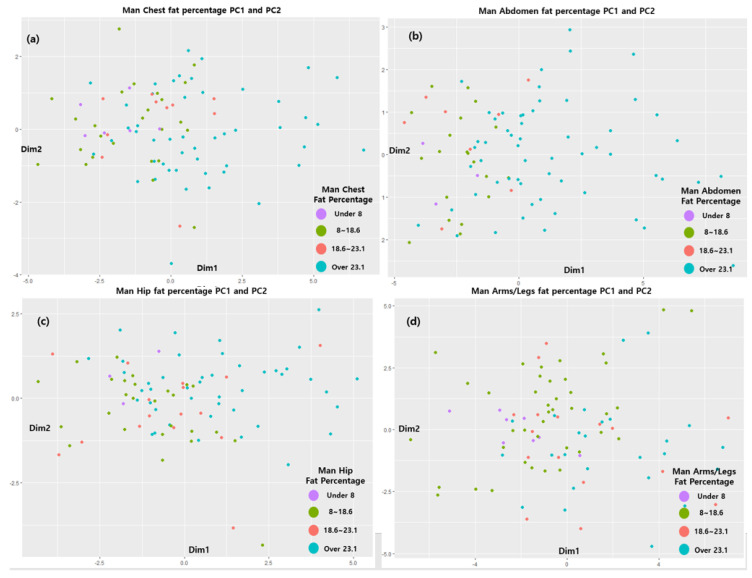
PC 1 and PC 2 results for (**a**) male chests, (**b**) male abdomens, (**c**) male hips, and (**d**) male arms-legs.

**Figure 6:**
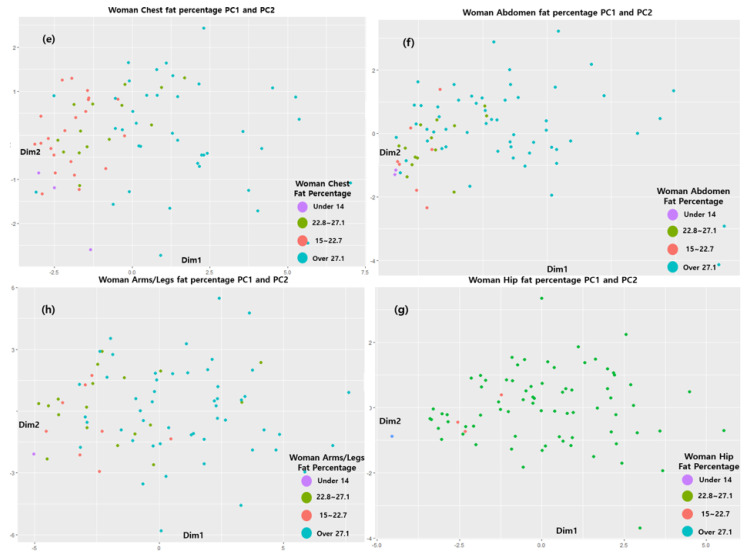
PC 1 and PC 2 results for (**e**) female chests, (**f**) female abdomens, (**g**) female hips, and (**h**) female arms-legs.

Therefore, to reduce the complexity of the model, we created a model using Comp 1 and Comp 2. The distribution is shown as a 2D graph. Therefore, as shown in Figure 4 and Figure 5, PCA models of the four regions were created for men and women, and the values corresponding to each distribution are shown. Figure 5 and Figure 6 and Table 6 and Table 7 show the distribution by region generated by the PCA model for men and women, respectively. PCA finds the axis with the largest variance in the training dataset and then finds the axis with the second largest variance that is orthogonal to the first axis. The third axis, which is orthogonal to the first and second axes, is also found in this way and can maximize the variance. In these processes, the unit vector is defined by finding the axes as many times as the number of dimensions of the dataset [34]. Therefore, one axis is called the principal component, and it is abbreviated as Comp.

## 4. Results

**Figure 7:**
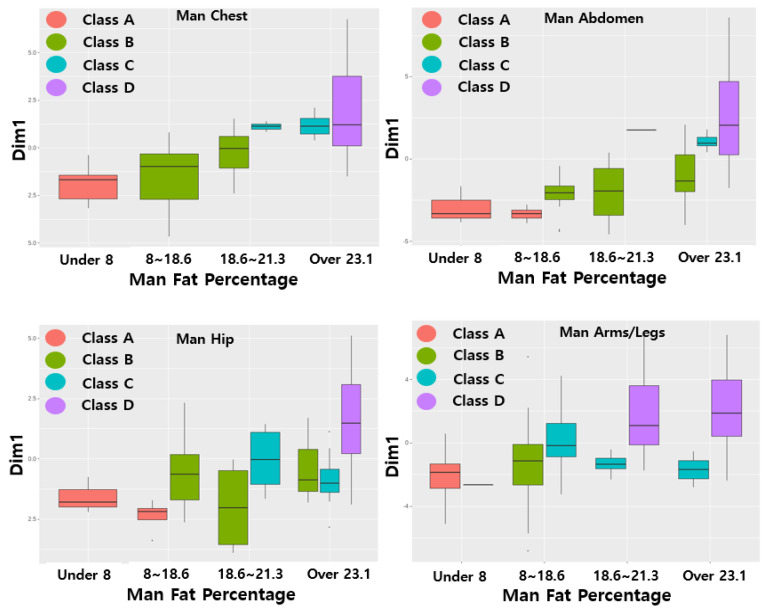

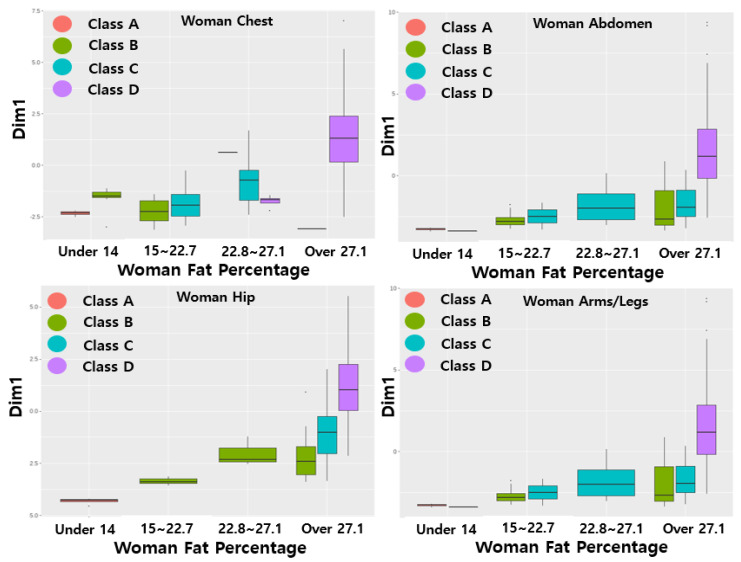
PCA value boxplots of classes A, B, C, and D (males-females).

Four classes based on the body fat percentage range were generated for each of the four body parts for males and females (men: under 8, 8–18.6, 18.7–23.1, and over 23.1; women: under 14, 15–22.7, 22.8–27.1, and over 27.1), and Comp 1 to Comp 5 were derived from the eigenvalue percentage of the variance of the PCA analysis for each of the four sites. The results showed that both men and women had more than 80% explanatory power for the data in Comp 1 and Comp 2 in the four areas. Therefore, a classification model for each body part was created using Type 1 and Type 2 data, which were derived from Comp 1 and Comp 2. This classification model was as follows: underweight→A, normal→B, overweight→C, and obese→D. The boxplot for the data group is shown in Figure 7.

Based on the generative model, when the body values for the four parts belonged to more than half of the class clusters for each body part because of clustering in the obese group based on the generative model, the measurer’s obesity information was added to the corresponding cluster (e.g., chest: A, abdomen: A, hips: A, legs/arms: B, obesity diagnosis: A) In addition, if the classes of two of the four regions were the same, the values corresponding to the higher class were used as the obesity information (e.g., chest: C, abdomen: C, hips: B, legs/arms: B, obesity diagnosis: C). However, this did not apply to all cases, and there were exceptions, as shown in Table 8.

**Figure 8:**
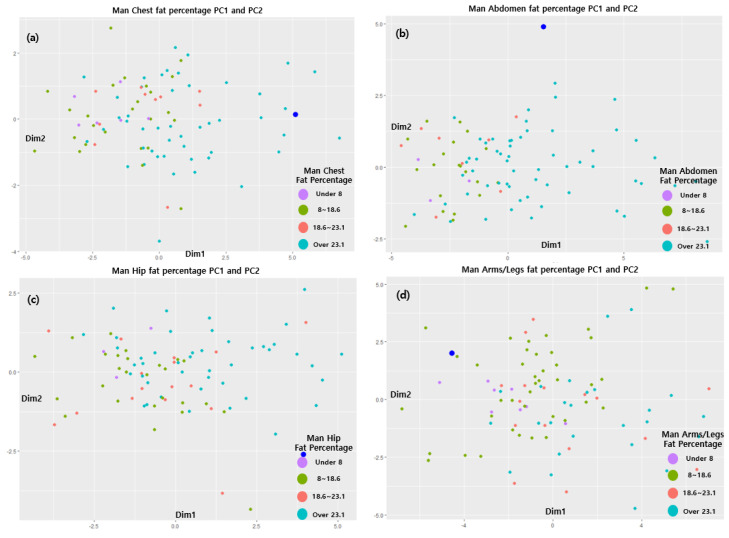
Validation results for the data generated.

Apart from the exceptions shown in Table 8, in all cases, it was possible to obtain obesity information from the cluster results for each of the four regions. Therefore, this model was validated based on the cluster results and for cases of exception. For verification, the model with the highest accuracy was selected using BMI, WHtR, and WHR, and the model proposed in this study was used for 10 random people for whom DEXA information and 3D body information (circumference, cross-sectional area, and volume) were available, and the results were compared with the existing obesity diagnosis indices. The body values of the group used for the verification of the proposed model are shown in Figure 8 as blue dots at the points where the PCA value for each body part belongs to the nearest class. Therefore, through a comparison of the distances of the classes corresponding to the obesity information, the class for each body part was derived as belonging to the class corresponding to the closest point.

The verification results are shown in Table 9. The proposed model showed high accuracy when judging the obese as follows: the D group and the underweight: A group for all obesity diagnosis models. However, in the case of the normal: B and overweight: C groups or the boundary between the obesity: D and the overweight: C groups, the obesity index values derived from a single measurement were not accurately judged. The verification of the model attained accuracy of 50% for BMI, 70% for WHtR, 60% for WHR, and 80% for the proposed model. Additionally, when comparing the overall obesity information judgment results, the BMI, which measures obesity by the relative height and weight, showed a low degree of purification, unlike other obesity diagnosis models, because information by body part and body type was not considered. Similarly, the WHtR, which uses waist circumference information, and the WHR, which utilizes hip circumference information, were found to have relatively low levels of accuracy compared with the model proposed in this study because they only use the information on specific areas. The model proposed in this study has a high diagnosis accuracy for identifying overweight and obesity. Additionally, unlike other diagnostic methods with low accuracy for identifying overweight and obesity, high accuracy was shown for overweight and obesity diagnoses. However, it was found to have similar or slightly better performance than the WHtR and WHR for underweight and normal diagnoses. Therefore, the model proposed in this study was shown to have high accuracy in terms of obesity diagnosis because its accuracy level was similar to that of DEXA for the diagnosis of overweight and obesity.

## 5. Conclusions and Future Research

This study utilized various body values (length, circumference, volume, and cross-sectional area) that can be derived from 3D data to determine the variables and regions that affect obesity and to measure obesity more precisely than existing obesity indices. Through a diagnosable model, guidelines for body information required for obesity diagnosis were presented. To this end, accurate fat percentage values were derived using a 3D scanner and a DEXA, which were designed based on ISO-7250 and can derive results for exact body points. Therefore, 3D body information about the chest, abdomen, buttocks, arms, and legs and the fat percentage and total fat percentage for each body part were used as learning data. For learning, data were collected from a total of 170 people (73 females and 87 males aged 20–30 years) from April to December 2021. For data collection, men wore tights as bottoms, and women wore tops and bottoms. The experimenter measured the total body fat percentage and the fat percentage for each body part through DEXA and derived body values using a 3D body scanner.

The analysis model created a model for each body part using the PCA model, which can identify the group characteristics of various variables. The body values of the arms and legs had strong positive correlations, so they were judged as one part, and the learning model was designed. Therefore, the following four PCA models were created for males and females: chest, abdomen, hips, and arms/legs. Based on the eigenvalue percentage of variance for the generated PCA model, four classes were created (underweight, normal, overweight, and obese), and the validation data were verified by randomly extracting data from 10 people who were assessed under the same conditions as used for the training data. For verification, the ground truth was compared with the three obesity indexes (BMI, WHtR, and WHR), which were existing obesity diagnosis methods, and the total fat percentage derived from the actual DEXA was used to determine the accuracy of the model proposed in this study. The model proposed in this study had an accuracy level of 80%, which was the highest accuracy level derived. Based on the results, the model proposed in this study was able to derive a 10–20% higher accuracy than the existing obesity indices when compared with DEXA, which derives the most accurate obesity information. As such, the method of deriving obesity information for each part using body values and diagnosing overall obesity using the obesity information for each part can provide more information than the existing method of diagnosing obesity simply by using one specific part. In addition, users can check whether they are at risk of obesity in any part of their whole body, even if their obesity information is normal. By using this information, users can manage their level of obesity and body shape and reduce body image distortion by obtaining accurate obesity information rather than obtaining obesity information using a specific number. In addition, many studies are being conducted to generate 3D body data using only images. By using this three-dimensional model of the body, not only can information about a body’s length, circumference, and volume be more easily obtained, but also, by using the model presented in this study, it is possible to accurately diagnose obesity without restrictions on location and equipment.

The method proposed in this study is one of the methodologies that can be used for accurate obesity diagnosis without DEXA information, and it aims to derive accurate obesity information using various types of body information. However, there were limitations in data collection for the group with excessive upper body development and lower body fat, and for this reason, it was not possible to accurately judge this group. In this study, detailed criteria for attaining general obesity information were presented, but people with special physical diseases or structures were not considered. In addition, exceptional treatment as shown in Table 9 was also performed for groups with special cases, but there is a limited ability to define all special groups. The reason why this group appeared is that there was a limited ability to evenly collect actual data for various groups. In future studies, it may be possible to accurately attain obesity information for groups with exceptional body values by recruiting more experimental groups and collecting data from groups with exceptional body values.

## Figures and Tables

**Table 1:** Variables derived from the 3D scanner and their descriptions (Body Landmarks: ISO-7250, Date: 5 April 2021–24 December 2021).

Collected Data	Explanation
Males (*n* = 87)	**Height (cm)** (Mean169.15/Max197/Min161) **Weight (kg)** (Mean 68.8/Max 120.6/Min 54.3) **BMI (kg/m^2^)** (Mean 23.35/ Max 16.6/Min 38.1)
Females (*n* = 73)	**Height (cm)** (Mean 162.8/Max 151/Min 175.7) **Weight (kg)** (Mean 56.6/Max 40.7/Min 89.6) **BMI (kg/m^2^)** (Mean 21/Max 16.2/Min 33.32)
**3D Scanner Variable Name**	**Explanation**
Height	Participant height
Weight	Participant weight
Volume	Participant body volume
Chest length	Shoulder height–chest height
Shoulder height	Height from the floor to the shoulders in standing position
Chest height	Height from the floor to the top of the nipples and the beginning of the chest in standing position
Breast height	Height from the floor to nipples in the standing position
Waist length	Waist height–hip height
Waist height	Height from the floor to the point in front of the waist in standing position
Belly button height	Height from standing position to the navel
Height below the belly button	Height from standing position to the iliac bone below the navel
Hip length	Hip height–groin
Hip height	Height from the floor to hip protrusion in standing position
Groin height	Vertical height from the floor to the groin (actual leg length)
Thigh length	Hip height–groin height
Thick thigh height	Height from the floor to the thickest part of the thigh
Mid-thigh height	Height from the floor to the mid-thigh
Knee height	Vertical height from the floor to the top of the shinbone
Calf height	Height from the floor to the point of the thickest part of the calf
Neck circumference	Circumference passing under the back of the neck and under the shield cartilage
Shoulder circumference	Circumference from the end of the shoulder to the end of the shoulder opposite the back of the neck
Chest circumference	Horizontal circumference through the midpoint of the sternum
Breast circumference	Horizontal circumference through the nipple point
Waist circumference	Horizontal circumference passing through the point in the front of the waist, the point in the side of the waist, and the point in the back of the waist
Belly waist circumference	Horizontal circumference passing through the navel point, the navel level, the waist point, the waist level, and the back point
Circumference below the belly button	Horizontal circumference through the iliac crest below the navel
Hip circumference	Horizontal circumference passing through the buttock protrusion
Groin circumference	Groin circumference
Thick thigh circumference	The circumference of the thickest part of the innermost thigh
Mid-thigh circumference	Circumference of the middle of the thigh
Knee circumference	Horizontal circumference through the midpoint of the kneecap
Calf circumference	Circumference of the most convex part of the calf
Arm circumference	Circumference of the thickest part of the upper arm with the arm raised
Cross-sectional area of the back of the neck	Cross-sectional area of the back of the neck
Shoulder cross-section	Cross-sectional area of the shoulder end point opposite the back neck point from the shoulder end point
Chest area	Cross-sectional area of the part that passes through the midpoint of the sternum
Breast area	Cross-sectional area of the part that passes through the nipple point
Waist area	Cross-sectional area of the part that passes through the front of the waist, the side of the waist, and the back of the waist
Navel waist area	Cross-sectional area of the part that passes through the navel point, the navel level, the waist point, the waist level, and the back point
Below the navel area	Cross-sectional area of the part that passes through the iliac crest below the navel
Hip area	Cross-sectional area of the part that passes through the buttock protrusion
Groin area	Cross-sectional area of the groin
Thick thigh area	Cross-sectional area of the thickest part of the innermost thigh
Median thigh area	Cross-sectional area of the middle part of the thigh
Knee area	Cross-sectional area passing through the midpoint of the kneecap
Calf area	Cross-sectional area of the most convex part of the calf
Total volume	Total body volume
Shoulder volume	Total volume at shoulder height
Chest volume	Total volume at chest height
Epigastric volume	Total volume corresponding to the upper abdomen
Lower abdominal volume	Total volume corresponding to the lower abdomen
Thigh volume	Total volume at thigh height
Calf volume	Total volume equivalent to calf height
Total abdominal volume	Upper abdominal volume + lower abdominal volume
**DEXA Variable Data**	**Explanation**
Chest Tissue	Percentage of fat in tissue (=Fat (g)/Tissue (g))
Android Tissue	Percentage of fat in tissue (=Fat (g)/Tissue (g))
Gynoid Tissue	Percentage of fat in tissue (=Fat (g)/Tissue (g))
Arm Tissue	Percentage of fat in tissue (=Fat (g)/Tissue (g))
Leg Tissue	Percentage of fat in tissue (=Fat (g)/Tissue (g))
Total Tissue	Percentage of fat in tissue (=Fat (g)/Tissue (g))

**Table 2:** Classification of obesity according to body fat percentage (total tissue %).

Obesity Class	Men	Women
Underweight	8% or less	14% or less
Normal	More than 8%, less than 18.6%	More than 14%, less than 22.7%
Overweight	More than 18.6%, less than 23.1%	More than 22.7%, less than 27.1%
Obese	More than 23.1%	More than 27.1%

**Table 3:** Three-dimensional scanner body values according to the fat percentage for each body part.

Body Part	Variables
Chest	Height, weight, chest length, shoulder area, chest area, breast area, shoulder volume, and chest volume
Abdomen	Height, weight, waist length, waist circumference, navel waist circumference, lower navel circumference, waist area, navel waist area, lower navel area, upper abdominal volume, and lower abdominal volume
Hips	Height, weight, hip length, hip circumference, groin circumference, hip area, and groin area
Arms/Legs	Height, weight, groin height, thigh length, knee height, calf height, thick thigh circumference, mid-thigh circumference, knee circumference, calf circumference, thick thigh area, mid-thigh area, knee area, calf area, and arm circumference

**Table 4:** Class values defined for each body part.

Men				
Body Part	Class 1	Class 2	Class 3	Class 4
Chest Abdomen Hips Arms/Legs	8% or less	More than 8%, less than 18.6%	More than 18.6%, less than 23.1%	More than 23.1%
**Women**				
**Body Part**	**Class 1**	**Class 2**	**Class 3**	**Class 4**
Chest Abdomen Hips Arms/Legs	14% or less	More than 14%, less than 22.7%	More than 22.7%, less than 27.1%	More than 27.1%

**Table 5:** PCA results.

Men					
Eigenvalue percentage of variance	Comp 1	Comp 2	Comp 3	Comp 4	Comp 5
Chest	63.8301	15.631	11.801	6.501	1.408
Abdomen	61.424	18.083	12.752	5.194	1.849
Hips	79.498	12.125	5.63	1.646	0.579
Arms/Legs	60.3	25.5	6.1	3.8	2.8
**Women**					
Eigenvalue percentage of variance	Comp 1	Comp 2	Comp 3	Comp 4	Comp 5
Chest	68.951	13.346	11.37	2.5	2.4
Abdomen	61.493	16.343	13.454	1.11	0.63
Hips	77.557	13.416	5.65	1.87	0.887
Arms/Legs	61.6	27.5	4.5	2.8	2.2

**Table 6:** Results for men.

Body Fat Percentage		Chest		Abdomen		Hips		Arms/Legs	
		Type 1	Type 2	Type 1	Type 2	Type 1	Type 2	Type 1	Type 2
Under 8	Mean	−1.97	0.25	−2.95	−0.46	−4.56	−0.87	−5.05	−2.08
	Median	−1.9	−0.01	−3.34	−0.48	−1.81	0.66	−2.25	0.05
	Min	−3.18	−0.17	−3.86	−1.16	−2.21	−0.16	−5.11	−1.04
	Max	−0.4	1.13	−1.67	0.27	−0.76	1.39	0.57	0.8
8–18.6	Mean	−1.39	0.1	−2.42	−0.14	−3.25	−0.54	−2.69	−0.61
	Median	−1	0.09	−2.32	−0.12	−1.17	−0.04	−0.89	0.76
	Min	−4.67	−2.7	−4.41	−2.06	−4.32	−4.34	−6.81	−2.63
	Max	0.81	2.76	−0.42	1.61	2.31	1.22	5.42	4.84
18.6–23.1	Mean	−0.5	0.15	−2.14	0.42	−2.03	−0.26	−1.79	0.27
	Median	−0.34	0.63	−2.46	0.86	−0.21	−0.45	−0.39	−0.001
	Min	−2.42	−2.66	−4.59	−1.74	−3.9	−3.83	−2.32	−3.99
	Max	1.52	0.96	0.37	1.76	4.02	1.57	7.02	3.48
Over 23.1	Mean	1.07	−0.11	1.28	0.01	0.14	0.02	0.15	0.04
	Median	0.57	−0.23	0.68	0.02	0.8	0.37	1.53	−0.97
	Min	−2.83	−3.69	−4.03	−2.6	−2.83	−1.96	−2.82	−4.71
	Max	6.75	2.16	8.59	2.94	5.1	2.62	6.79	3.9

**Table 7:** Results for women.

Body Fat Percentage		Chest		Abdomen		Hips		Arms/Legs	
		Type 1	Type 2	Type 1	Type 2	Type 1	Type 2	Type 1	Type 2
Under 14	Mean	−2.29	−1.54	−3.4	−1.22	−4.56	−0.87	−5.05	−2.08
	Median	−2.71	−1.196	−3.4	−1.22	−3.85	−0.75	−4.79	−1.52
	Min	−3.01	−2.59	−3.41	−1.3	−4.56	−0.87	−5.05	−2.08
	Max	−1.35	−0.85	−3.38	−1.15	−3.15	−0.64	−4.53	−0.97
15–22.7	Mean	−1.97	0.03	−2.53	−0.7	−3.25	−0.54	−2.69	−0.61
	Median	−1.96	−0.04	−2.57	−0.88	−2.32	−0.22	−2.71	0.19
	Min	−3.13	−1.33	−3.3	−2.33	−2.88	−0.8	−3.19	−2.92
	Max	−0.25	1.29	−1.67	1.39	−0.53	1.48	−2.29	2.91
22.8–27.1	Mean	−0.88	0.26	−1.93	−0.34	−2.03	−0.26	−1.79	0.27
	Median	−1.36	0.165	−2.21	−0.42	−1.45	−0.08	−1.37	−0.21
	Min	−2.34	−1.14	−3.25	−1.85	−3.4	−1.14	−4.84	−2.61
	Max	1.68	1.3	0.14	0.87	2	0.85	3.32	1.95
Over 27.1	Mean	1.6	0.01	0.94	0.22	0.14	0.02	1.15	0.04
	Median	1.29	0.082	0.59	0.14	0.72	−0.01	1.1	0.25
	Min	−3.09	−2.72	−3.37	−4.13	−3.35	−3.69	−4.5	−5.81
	Max	7.02	2.43	9.37	3.22	5.53	3.35	7.63	5.48

**Table 8:** Exceptions by group for males and females.

Obesity Diagnosis	Chest	Abdomen	Hip	Legs/Arms	Obesity
Male	C	D	D	C	Overweight
Male	A	B	B	B	Normal
Male	B	C	B	B	Normal
Male	D	D	B~C	B~C	Overweight
Male	A~B	C~D	A~B	A~B	Normal
Female	B~C	D	D	D	Overweight
Female	B	C~D	D	C~D	Overweight

**Table 9:** Results obtained with the obesity diagnosis model.

Obesity Diagnosis	DEXA	BMI	WHtR	WHR	Proposal Model
Sample 1	D	D	D	C	D
Sample 2	C	B	C	C	C
Sample 3	B	B	B	B	B
Sample 4	C	B	C	C	C
Sample 5	C	B	C	B	C
Sample 6	B	A	A	B	B
Sample 7	D	C	C	C	C
Sample 8	A	A	B	B	A
Sample 9	A	A	A	A	B
Sample 10	B	B	B	B	B
Accuracy	Ground Truth	50%	70%	60%	80%

## Data Availability

The data is personal data for experimentation purposes and will not be reported.
